# Supplementary material for: Progestin-primed ovarian stimulation for fertility preservation in women with cancer: A comparative study
Source: PLoS One. 2023 Mar 28;18(3):e0280238. doi: 10.1371/journal.pone.0280238 (PMC10047511; doi:10.1371/journal.pone.0280238)
Supplement: Attachment — Submitted filename: Filippi et al., Response to reviewers.doc [file pone.0280238.s002.doc]

**Response to reviewers and Editorial office**

*1. Please ensure that your manuscript meets PLOS ONE's style requirements, including those for file naming. The PLOS ONE style templates can be found at* 
[*https://journals.plos.org/plosone/s/file?id=wjVg/PLOSOne_formatting_sample_main_body.pdf*](https://journals.plos.org/plosone/s/file?id=wjVg/PLOSOne_formatting_sample_main_body.pdf) *and*
[*https://journals.plos.org/plosone/s/file?id=ba62/PLOSOne_formatting_sample_title_authors_affiliations.pdf*](https://urlsand.esvalabs.com/?u=https%3A%2F%2Fjournals.plos.org%2Fplosone%2Fs%2Ffile%3Fid%3Dba62%2FPLOSOne_formatting_sample_title_authors_affiliations.pdf&e=19a35edd&h=f0bbe76a&f=n&p=y)

REPLY: Done

*2. Thank you for stating the following financial disclosure:*

*"This study was partially funded by Italian Ministry of Health – Current research IRCCS."*

*At this time, please address the following queries:*

*a) Please clarify the sources of funding (financial or material support) for your study. List the grants or organizations that supported your study, including funding received from your institution. 
b) State what role the funders took in the study. If the funders had no role in your study, please state: “The funders had no role in study design, data collection and analysis, decision to publish, or preparation of the manuscript.”
c) If any authors received a salary from any of your funders, please state which authors and which funders.
d) If you did not receive any funding for this study, please state: “The authors received no specific funding for this work.”*

*Please include your amended statements within your cover letter; we will change the online submission form on your behalf.*

REPLY: Done

*3. Thank you for stating the following in the Competing Interests section:*

*"**I have read the journal's policy and the authors of this manuscript have the following competing interests: E.S. received honoraria for presentations at meetings from Gedeon-Richter and Merck-Serono. He is also handling two grants of research from Ferring. None of the remaining authors reported any conflict of interest."*

*Please confirm that this does not alter your adherence to all PLOS ONE policies on sharing data and materials, by including the following statement: "**This does not alter our adherence to  PLOS ONE policies on sharing data and materials.” (as detailed online in our guide for authors* [*http://journals.plos.org/plosone/s/competing-interests).*](https://urlsand.esvalabs.com/?u=http%3A%2F%2Fjournals.plos.org%2Fplosone%2Fs%2Fcompeting-interests).%26nbsp%3B&e=19a35edd&h=8ba961c6&f=n&p=y)  *If there are restrictions on sharing of data and/or materials, please state these. Please note that we cannot proceed with consideration of your article until this information has been declared.*

*Please include your updated Competing Interests statement in your cover letter; we will change the online submission form on your behalf.*

REPLY: Done

*4. Please review your reference list to ensure that it is complete and correct. If you have cited papers that have been retracted, please include the rationale for doing so in the manuscript text or remove these references and replace them with relevant current references. Any changes to the reference list should be mentioned in the rebuttal letter that accompanies your revised manuscript. If you need to cite a retracted article, indicate the article’s retracted status in the References list and also include a citation and full reference for the retraction notice.*

REPLY: Done

**Reviewers' comments:
Reviewer's Responses to Questions**

**Comments to the Author**

*1. Is the manuscript technically sound, and do the data support the conclusions?
The manuscript must describe a technically sound piece of scientific research with data that supports the conclusions. Experiments must have been conducted rigorously, with appropriate controls, replication, and sample sizes. The conclusions must be drawn appropriately based on the data presented.*

*Reviewer #1: Partly*

*Reviewer #2: Yes*

REPLY: We thank the reviewers for appreciating our manuscript and we agree on the general judgments (albeit not univocal).

*2. Has the statistical analysis been performed appropriately and rigorously?*

*Reviewer #1: Yes*

*Reviewer #2: Yes*

REPLY: Thank you again for the appreciation.

*3. Have the authors made all data underlying the findings in their manuscript fully available?
The* [*PLOS Data policy*](https://urlsand.esvalabs.com/?u=http%3A%2F%2Fwww.plosone.org%2Fstatic%2Fpolicies.action%23sharing&e=19a35edd&h=68781fa2&f=n&p=y) *requires authors to make all data underlying the findings described in their manuscript fully available without restriction, with rare exception (please refer to the Data Availability Statement in the manuscript PDF file). The data should be provided as part of the manuscript or its supporting information or deposited to a public repository. For example, in addition to summary statistics, the data points behind means, medians and variance measures should be available. If there are restrictions on publicly sharing data—e.g., participant privacy or use of data from a third party—those must be specified.*

*Reviewer #1: Yes*

*Reviewer #2: Yes*

REPLY: Thank you again

*4. Is the manuscript presented in an intelligible fashion and written in standard English?
PLOS ONE does not copyedit accepted manuscripts, so the language in submitted articles must be clear, correct, and unambiguous. Any typographical or grammatical errors should be corrected at revision, so please note any specific errors here.*

*Reviewer #1: Yes*

*Reviewer #2: Yes*

REPLY: Thank you again

*5. Review Comments to the Author
Reviewer #1:*

*The title of the manuscript sounds confusing. It will benefit if rephrased for more clarity.*

REPLY: The tile has been changed. It is now “Progestin-primed ovarian stimulation for fertility preservation in women with cancer: a comparative study**”**

*The abstract provides some understanding of the study, however, should be improved to represent the study in a clearer way. In the methods section of the abstract, the study design should be explained. It is not clear what does "before and after '' mean?*

REPLY: We agree on this criticism. The abstract was not clear. We have extensively revised it. We have removed the terms “before and after” and we have deleted the part on the concentration of sex steroids in the follicular fluids (that was a corollary finding of the study). We believe it is now much more understandable.

*The manuscript draft is landscape oriented. Is there any practical need for it? Please follow the PLOS ONE journal requirements at* [*https://journals.plos.org/plosone/s/submission-guidelines*](https://urlsand.esvalabs.com/?u=https%3A%2F%2Fjournals.plos.org%2Fplosone%2Fs%2Fsubmission-guidelines&e=19a35edd&h=af2ab1dc&f=n&p=y)*.*

REPLY: Sorry for the mistake. Now changed.

*The introduction part provides a clear rationale for the study. However, it is too narrow, and the text will benefit if reworked. In particular, more updated references are suggested. For example for the sentences in lines 39-41 and 42-46 the following more updated sources could be used - doi: 10.3332/ecancer.2020.1030; doi: 10.3390/jcm11010196; doi: 10.3390/ijms222111825; doi: 10.1007/s00404-018-5006-z.*

REPLY: As suggested, we have added the suggested citations (except for the last one that focussed on elderly women - 10.1007/s00404-018-5006-z). We did it, since requested, but we disagree on the criticism that our references were not updated (we cited all recent guidelines on fertility preservation). Moreover, we did not think it was necessary to touch the topic of endometrial cancer. However, as mentioned earlier, this has now been done. Moreover, the introduction has been extended.

*Moreover, information on the epidemiology of GYN cancers in general and among young women and the current situation in your country with the fertility preservation approach could improve the introduction part.*

REPLY: As mentioned in the previous point, our study does not focus on gynaecological cancers (in fact, many gynaecological cancers such as advanced endometrial cancer or cervical cancer do not benefit from oocytes cryopreservation). In our opinion, discussing specifically gynaecological cancers would not improve the clarity of the introduction. We have just mentioned to endometrial cancer (see previous point) only because this is a good example to highlight the growing relevance given to preserving fertility in modern oncology.

We also do not agree on focussing on the situation in Italy. This study aims at providing messages that could be valid anywhere. For this reason, the main citations used in the introduction (Oktay *et al*., 2018; **Practice Committee of ASRM, 2019;** ESHRE Guideline Group on Female Fertility Preservation, 2020; Lambertini *et al*., 2020) refer to guidelines of the most relevant scientific societies with interest in the field, two from US and two from Europe (ASCO, ASRM, ESHRE and ESMO).

*The methods section is detailed. However, poorly structured. Please provide the following items (better to highlight with the relevant subheadings, which will make the text more comprehensible for a potential audience): Study Design,* *study participants/subjects, Study instruments, variables, study protocol,* *ethical considerations, and* *statistical analyses.*

REPLY: As requested, subheadings have been added.

*The text in lines 107-114 of the methods section looks confusing. It might sound better if moved to the results section. However, it is up to the authors to decide.*

REPLY: Lines 107-114 are the figure legend of Figure 1. They must be read with the figure in front. We have moved this legend to a better place to facilitate comprehension. Moreover, we have modified some part to improve clarity.

*The results are promising and presented clearly, supported by tables and figures.*

REPLY: Thank you for appreciating our presentation

*The discussion part is interesting but will improve if restructured according to the suggested outline (the following items should be mentioned**): Rationale of the study (why it was done); Main findings of the study; What makes your study unique;* *What it adds to what we already know;* *Subject of the discussion,* *Comparison with previous studies, agreement and disagreement with the studies compared; study* *strengths and limitations;* *Clinical implication.*

REPLY: The stringent application of this schematic presentation may not be always valid. However, since suggested by the reviewer, we have tried to force our discussion into this scheme. We have also added sub-headings (but we have no concerns if the publisher will decide to remove them).

***Reviewer #2:***

*I read with great interest the manuscript, which falls within the aim of this Journal. In my honest opinion, the topic is interesting enough to attract the readers’ attention. Nevertheless, the authors should clarify some points and improve the discussion, as suggested below.*

REPLY: We thank the reviewer for appreciating our efforts.

*Authors should consider the following recommendations:
- I recommend to discuss, at least briefly, novel pieces of evidence about fertility-preservation in case of early-stage endometrial cancer, since it is one of the most common gynecological malignancies in women of reproductive age (authors may refer to: PMID: 36143933).*

REPLY: As mentioned to the reply of the first reviewer, the present study is not focussed on gynecological cancer, not even on endometrial cancer. Noteworthy, endometrial cancer is a rare indication to egg freezing. On the other hand, we agree that endometrial cancer is an outstanding symbol of fertility sparing philosophy. Therefore, a comment on this cancer has been added in the introduction

*- Authors may consider to add further details about the psychological impact of cancer in women wishing to preserve their fertility (PMID: 28275393)*

REPLY: Good suggestion. Done
